# The Effect of the COVID-19 Pandemic on Emergency Department (ED) Admissions in the Only Hospital of City Center ED

**DOI:** 10.7759/cureus.44527

**Published:** 2023-09-01

**Authors:** Bedriye Feyza Kurt, Oya Güven, Hakan Selçuk

**Affiliations:** 1 Emergency Department, Kırklareli Training and Research Hospital, Kırklareli, TUR

**Keywords:** triage, pandemic, emergency department, covıd-19, admission rate

## Abstract

Aim: This study aimed to examine the effect of the pandemic on emergency service visits, together with the pre- and post-pandemic period data.

Material and methods: The charts of patients who applied to the emergency department between 2019 and 2021 were included in the study. We analysed patients' charts from the pre-pandemic period (January 1, 2019-February 29, 2020), the pandemic period (March 1, 2020-June 30, 2021; from the date of detection of the first COVID-19 case to the date of the second dose of the vaccine), and normalisation period (July 1, 2021-December 31, 2021; the date from the completion of vaccination to the end of the year). Demographic characteristics, triage codes, diagnoses, hospitalisation or referral status, population ratio, admission rate, and mortality were examined in these data.

Results: In total, 529,706 patient charts were examined. When the pre-pandemic period (15,983.29±1,493.19) was compared with the pandemic period (11,342.94±2,350.15), it was observed that there was a decrease in the number of visit period. In the post-pandemic period, patients coming to the hospital decreased following vaccination. It was determined that there were more visits (20742.17±967.61) compared to the pre-pandemic period.

Conclusion: The data demonstrate that, during the pandemic period, visits decreased in general, and the rate of critical patients increased gradually. Accordingly, there are unnecessary visits and inappropriate use of emergency services.

## Introduction

The World Health Organization (WHO) declared that the novel coronavirus disease 2019 (COVID-19), which was first detected in Wuhan, China, has become a global health concern, causing severe respiratory tract infections in humans [1].

Turkey announced the first coronavirus case on March 11, 2020. After that, the government applied many preventive measures, such as stay-at-home orders (such as travel restrictions, closure of schools and public places, and a curfew under the age of 20 and over 65). Considering the decrease in the number of cases and daily deaths and the corresponding recovery rates, some restrictions were lifted in June 2020, and in July, a controlled social life period started. According to this, Turkey's first significant peak in COVID-19 cases occurred between March and May 2020. In January 2021, widespread vaccination started and triggered the relaxation of restrictions [2,3].

The impact of COVID-19 on the economic and medical systems is significant. Overcrowding in the emergency department (ED) is an important worldwide public health and healthcare problem. The biggest reason why emergency services are crowded in Turkey is non-emergency patient admissions [4]. EDs are on the front lines during the COVID-19 pandemic and have been the most affected department in hospitals. EDs are outpatient clinics where COVID-19 patients are admitted in most hospitals. In contrast to the default state of overcrowded healthcare systems, statistics from other countries have shown a reduction in the ED patient volume early in the COVID-19 pandemic [5]. It has been shown that, in some studies, the patterns of ED visits also changed significantly during the pandemic season [6-8]. However, most of these studies were planned from data from the first year of the fight against COVID-19. The literature does not have enough data on when the vaccine became widespread and its effects were seen.

This study analysed patients' charts who came to the ED between 2019 and 2022 in this way tried to investigate the impact of COVID-19 on the ED compared to the pre-pandemic and post-pandemic periods. Thus, this study's results may contribute to public health planning.

## Materials and methods

Ethical statement

The Kırklareli University Scientific Research Ethics Committee (P20220002) and the Ministry of Health COVID-19 study platform (2022-01-14T13_56_38) have approved this study.

Patient selection

The study retrospectively reviewed the charts of all patients admitted to the only hospital in the city center EDs for any reason between 2019 and 2022. Demographic characteristics, triage codes (green, yellow, red), mode of arrival (ambulance, not admitted), primary diagnoses, intensive care service and hospitalisation rates, and mortality were examined. The charts have been analysed from the archive using the electronic registration system of the hospital. Patients admitted to the ED were evaluated according to the Ministry of Health Triage rules. According to the rules, “red” is for unstable urgent, “yellow” is for acute, and “green” is for non-urgent [9].

Time range

Patient visits were classified based on March 2020, when the first case of COVID-19 was seen in our country. The one year before this date was considered the pre-pandemic period until July 2021; when the second dose of vaccination was completed, the pandemic; and the period until the end of the year, when quarantine practices were stretched, was considered the normalisation period.

Pre-pandemic period: From 1 January 2019 to 29 February 2020 (14 months)

Pandemic period: From 1 March 2020 to 30 June 2021 (16 months)

The period when the effect of the pandemic decreased (normalization period): From 1 July 2021 to 31 December 2021 (six months) 

The charts were examined according to this range.

Statistical analysis

Statistical analyses were performed using Statistical Product and Service Solutions (SPSS) version 19.0 for Windows (IBM SPSS Statistics for Windows, Armonk, NY). Descriptive criteria are presented as mean, standard deviation, and percentage distribution. The conformity of the data to the normal distribution was checked with the Kolmogorov-Smirnov test. Kruskal-Wallis analysis and Mann-Whitney U test were used to compare the mean numbers of various features between time groups. In cases that were statistically significant in the Kruskal-Wallis analysis, the Mann-Whitney U analysis was admitted for post-hoc analysis, and the Bonferroni-corrected p-value was taken as 0.017. The significance level was taken as p<0.05.

## Results

In this study, 529,706 patient charts were examined. Of these, 251,809 (47.53%) were female, and 277,897 (52.46%) were male. Female and male patient numbers were close to each other before the pandemic and during the normalisation period, while the number of male patients was dominant during the pandemic. The mean age of the patients increased during the pandemic period. It was observed that the average number of visits was higher in the normalisation period compared to other periods (Table 1).

**Table 1:** Disease rates by period GIS: Gastrointestinal system

Diagnosis Rate (%) Time period (n=Total number)	Skin	Cardiac	Endocrine	Respiratory	Obstetric	Neoplasm	GIS	Trauma	Neurology
Before Pandemic (n=223,766)	1.74	20.72	0.30	17.69	0.10	0.01	6.60	25.25	1.83
Pandemic (n=181,487)	2.29	22.01	0.29	18.94	0.07	0.01	4.59	26.42	1.74
Normalized Period (n=124,453)	1.83	23.09	0.22	21.26	0.09	0.04	5.95	26.41	1.18
Total	2.0	21.6	0.28	18.85	0.08	0.02	5.60	25.96	1.68

The admission rate of cardiac, respiratory, and trauma patients has increased. The number of gastrointestinal system disease (GIS) patients decreased during the pandemic, but the number of skin disease patients increased (Table 2).

**Table 2:** Comparison of the modes of arrival, inpatient number, referred, and resuscitation ICU: Intensive care unit CPR: Cardiopulmonary resuscitation

	Before Pandemic (n=223,766)	Pandemic (n=181,487)	Normalized period (n=124,453)	p-value
Mode of arrival: Walk-in	212,688 (95%)	166,368 (91.6%)	118,669 (95.3%)	
Mode of arrival: Ambulance	11,078 (5%)	15,119 (8.3%)	5,784 (4.7%)	0.001
Inpatient (Total 12,326), Clinic	n=4,060	n=5,545	n=2721	
	3,376 (83.1%)	4,403 (79.4%)	2,178 (80%)	
ICU	684 (18.9%)	1,142 (20.6%)	543 (20%)	0.004
Referred to other hospitals	1,139 (0.50%)	1,000 (0.55%)	406 (0.32%)	0.024
Number of patients, CPR	148 (0.06%)	231 (0.12%)	99 (0.07%)	0.064

**Figure 1:**
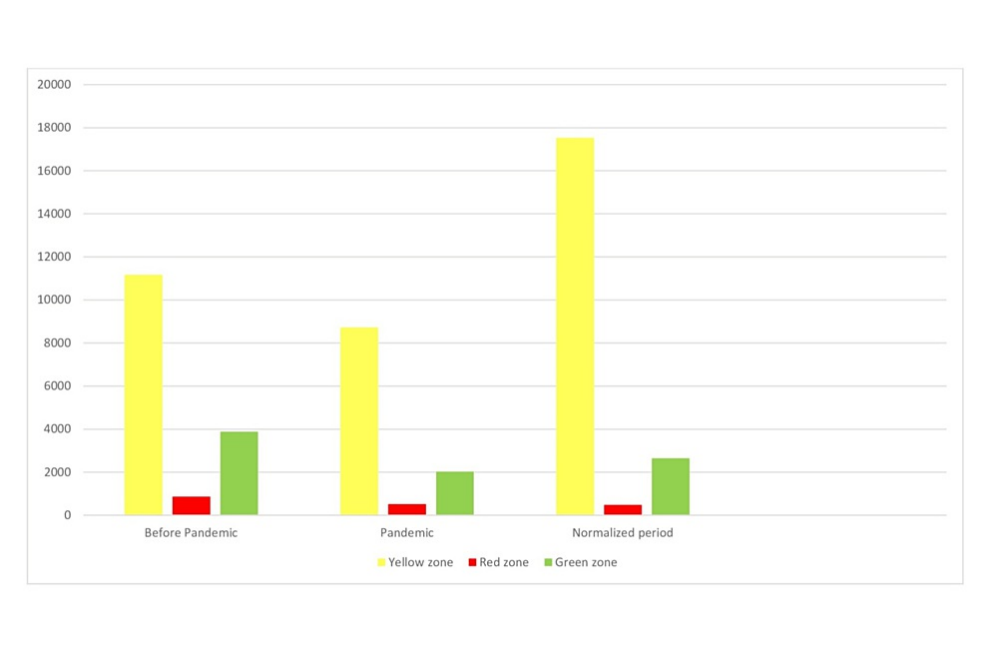
Triage rates by periods

When the triage codes were examined according to the urgency of the patients, the number of patients with yellow codes in all periods was higher than the others. It was determined that the patients with green and red codes during the normalisation period did not reach the numbers in the pre-pandemic period (Figure 1).

During the pandemic, the number of admissions by ambulance, inpatients, and those who underwent cardiopulmonary resuscitation (CPR) increased. In the normalisation period, the number of outpatients was higher than that in the previous periods (Table 3).

**Table 3:** Population, total number of applications, and mortality rate

	2019	2020	2021
Population (n)	361,836	361,737	366,363
Total number of applications (n)	188,920	142,683	198,103
Rate (%)	52.2	39.4	54
Mortality (n, %)	555 (0.29)	441 (0.30)	438 (0.22)

In the study, although the population did not change significantly, it was observed that admissions decreased relatively during the pandemic period. Still, the mortality rate (compared to admitted patients) increased (Table 3).

## Discussion

This study tried to examine how much the emergency services, which are the first place patients apply, are affected in events that affect communities, such as pandemics, through the emergency service data of a single hospital in the city centre. This study is the first to include data on emergency room admissions for the post-pandemic normalisation period. Further, no previously published literature was found on the use of ED after vaccination.

In the study, it was determined that, during the pandemic period, male patients were admitted to the emergency service more than female patients, and the average age of the patients was high. According to this, it can be said that the elderly population, which is a sensitive population to COVID-19, admitted more. In addition, considering that the female population, which obeys the hygiene rules more, is protected from the disease, this result is significant [10].

Several studies have reported similar results, with a sharp decrease in ED visits during the pandemic [11,12]. Similarly, this study observed that, at the beginning of the pandemic, the number of ED visits decreased rapidly with the announcement of deaths and quarantine. This decrease was because people avoided ED visits for non-COVID-related illnesses and delayed their visits as long as possible because of the pandemic and possibly patients' fears of contracting COVID-19 [13].

The fact that social distancing measures have reduced the spread of infection along with the quarantine may also be a factor in the decrease in ED visits. Hospitals cancelled elective surgeries, resulting in more emergency room visits due to complications [14,15]. In addition, patients who required strict follow-up were not followed up, so they deteriorated rapidly, and the death rate may have increased due to all of these [16]. The increase in the number of oncology patients in the post-pandemic period compared to the past corroborates this conclusion.

Health professionals perform colour-coded triage in emergency services for effective service delivery. Generally, green-coded patients are given a prescription, yellow-coded patients are given short-term symptomatic treatment in the hospital (or inpatient treatment in the ward), and red-coded patients are the patient group who receive intensive care follow-up or are mortal. According to the findings obtained in this study, the number of patients with all colour codes decreased significantly during the pandemic period, but the number of patients with a yellow code increased considerably in the post-pandemic period. The visits of GIS patients have been an excellent example of this situation. Due to the acute development of GIS symptoms and disrupting patient comfort, emergency services are the first-choice outpatient clinic. This type of patient decreased during the pandemic and increased again close to the old rate during normalisation. Generally, the rate of green-coded patients in emergency services is higher than that in others. However, according to the results of this study, it can be said that the main burden of emergency services is yellow-coded patients. Similar results were found in a study conducted in a region with few hospitals, as in our city [17]. The reason for this may be that IV treatment is frequently given because there is no other emergency service that patients can apply to, and therefore the triage code of the patients is changed to yellow even if it is green.

Considering studies from many countries, COVID-19 affected hospitalisations and ED admissions. In a study conducted by Wongtanasarasin et al., during a national lockdown for COVID-19, there was a significant reduction in daily ED visits. However, it was determined that the rates of hospitalisation and follow-up in the intensive care unit increased [18]. This suggested a potential delay in the presentation. In this study, findings similar to the literature were obtained. In addition, the increase in the number of patients who underwent CPR or were followed up in the intensive care unit during the pandemic suggests that the patient's clinical condition deteriorated rapidly due to infection.

In this study, when the patients were examined according to the form of visits, it was observed that the rate of outpatients decreased during the pandemic period and increased more than before after the pandemic. This situation can be expected during the normalisation period, but the increase in the number more than before can be called the 'new normal period'. It was determined that the number of patients arriving by ambulance increased during the pandemic period. In our province, during the quarantine period, patients with PCR+ or COVID-19 symptoms were transferred to the hospital by ambulance for isolation, regardless of the triage code. In addition, although there is a notable decrease in the number of patients with other heart diseases, such as atrial fibrillation and heart failure, according to this study’s results, the unchanged proportions of patients with severe conditions, such as acute myocardial infarction, trauma, childbirth, endocrine emergencies, indicate that 112 is still called during quarantine. These cases in the code red category usually arrive by ambulance and are the accurate indication of their emergency admission. However, interestingly, it has been determined that skin diseases and visits to the ED have increased during the pandemic period. The most important factor affecting this situation may be the epidemic of scabies [19].

During the pandemic period, since the hospital where the study was conducted was declared the only pandemic hospital in the province, it only served patients with COVID-19 infection. This explains why the referral rate in this period is higher than in other periods.

In our country, the COVID-19 vaccine started to be administered with the inactivated vaccine (Sinovac-CoronaVac Vero Cell) in January 2021 and continued with the m-RNA vaccine (BioNTech) in April [20]. The second dose of vaccination was completed in July 2021. In the period from July 2021 to the end of the year, although a very short period of six months passed, it was observed that the number of visits exceeded that of the pre-pandemic period. Moreover, when looking at the population and the annual average number of visits in our province, it has been determined that the visits have significantly decreased during the pandemic, but increased more than before during the normalisation period. It can be thought that the fear of infection has reduced with the confidence of being protected from the disease, and emergency service visits have increased again.

Emergency services are the first place of visit for patients because of the perception that patients can be seen without an appointment, and consultations from the emergency department are quick for patients who cannot get an appointment at the relevant branch. Because of this crowd, the emergency service team is prepared for any chaotic scenario [4].

It has been observed that the burden of emergency services has increased even more due to the rapid spread and high number of cases all over the world during the pandemic process. In addition, this study is a representative example of how the emergency services in our country serve a comprehensive patient profile and how high the workload is. Accordingly, emergency services must be prepared to face the challenges of the next pandemic or other infectious diseases. With such studies, it can be shown that emergency services are exposed to unnecessary visits.

## Conclusions

This study has shown that there has been a severe decrease in ED visits due to the lockdowns at the onset of the pandemic and people's fear of exposure to COVID-19. However, this situation has started to go back to the past with the spread and the effect of vaccination in 2021. The results strongly suggest that COVID-19 significantly affected people's behaviour toward the unnecessary use of EDs.

## References

[1] Zhai P, Ding Y, Wu X, Long J, Zhong Y, Li Y (2020). The epidemiology, diagnosis and treatment of COVID-19. Int J Antimicrob Agents.

[2] (2022). Turkiye’de Covid-19 pandemisi zaman cizelgesi. https://tr.wikipedia.org/wiki/Turkiye%E2%80%99de_Covid-19_pandemisi_zaman_cizelgesi.

[3] T.C. Sağlık Bakanlığı-Koronavirüs-bilim-toplantısına-ilişkin-açıklama-05.01.22. https://www.saglik.gov.tr (2022). Koronavirüs bilim kurulu toplantısına İlişkin açıklama (05.01.2022). https://www.saglik.gov.tr/TR,87061/koronavirus-bilim-kurulu-toplantisina-iliskin-aciklama-05012022.html.

[4] Kilicaslan I, Bozan H, Oktay C, Göksu E (2005). Türkiye’de acil servise başvuran hastaların demografik özellikleri. Türkiye Acil Tıp Dergisi.

[5] Baugh JJ, White BA, McEvoy D, Yun BJ, Brown DF, Raja AS, Dutta S (2020). The cases not seen: patterns of emergency department visits and procedures in the era of COVID-19. Am J Emerg Med.

[6] Çıkrıkçı Işık G, Tandoğan M, Şafak T, Çevik Y (2020). Retrospective analyses of the frequent emergency department users. Eur J Emerg Med.

[7] Lai YW, Hsu CT, Lee YT, Chen WL, Chen JH, Huang CC, Chung JY (2021). Analysis of COVID-19 pandemic impact on the presenting complaints of the emergency department visits. Medicine (Baltimore).

[8] Lo HY, Chaou CH, Chang YC, Ng CJ, Chen SY (2021). Prediction of emergency department volume and severity during a novel virus pandemic: experience from the COVID-19 pandemic. Am J Emerg Med.

[9] T.C. Sağlık Bakanlığı-Yataklı Sağlık Tesislerinde Acil Servis Hizmetlerinin Uygulama Usul Ve Esasları Hakkında (2022). Sağlık Bakanlığı-Yataklı Sağlık Tesislerinde Acil Servis Hizmetlerinin Uygulama Usul Ve Esasları Hakkında. https://www.mevzuat.gov.tr/mevzuat?MevzuatNo=39719&MevzuatTur=9&MevzuatTertip=5.

[10] Altun Y (2020). Covid-19 pandemisinde kaygı durumu ve hijyen davranışları. Sürekli Tıp Eğitimi Dergisi.

[11] Kuitunen I, Ponkilainen VT, Launonen AP, Reito A, Hevonkorpi TP, Paloneva J, Mattila VM (2020). The effect of national lockdown due to COVID-19 on emergency department visits. Scand J Trauma Resusc Emerg Med.

[12] Slagman A, Behringer W, Greiner F (2020). Medical emergencies during the COVID-19 pandemic. Dtsch Arztebl Int.

[13] Fung TH, Kuet M, Patel MK, Puri P (2020). Addressing COVID‐19 fear to improve clinic attendance for patients with wet age‐related macular degeneration. Acta Ophthalmol.

[14] Perea del Pozo E, Aparicio-Sánchez D, Hinojosa Ramírez F (2020). A prospective cohort study of the impact of covid19 world pandemic on the management of emergency surgical pathology. Br J Surg.

[15] Ciarleglio FA, Rigoni M, Mereu L (2021). The negative effects of COVID-19 and national lockdown on emergency surgery morbidity due to delayed access. World J Emerg Surg.

[16] Wong LE, Hawkins JE, Langness S (2020). Where are all the patients? Addressing Covid-19 fear to encourage sick patients to seek emergency care. Nejm Catalyst.

[17] Çevik C, Tekir Ö (2014). Acil Servis Başvurularinin Tani Kodlari, Triyaj Ve Sosyo-Demografik Açidan Değerlendirilmesi. Balıkesir Sağlık Bilimleri Dergisi.

[18] Wongtanasarasin W, Srisawang T, Yothiya W, Phinyo P (2021). Impact of national lockdown towards emergency department visits and admission rates during the COVID-19 pandemic in Thailand: a hospital-based study. Emerg Med Australas.

[19] Turan Ç, Metin N (2021). Impact of pandemic in the frequency of scabies: possible scabies outbreak scenario aftermath COVID-19. Turkiye Parazitol Derg.

[20] (2022). Turkiye’de Covid-19 asilamasi. https://tr.wikipedia.org/wiki/Turkiye%E2%80%99de_Covid-19_asilamasi.

